# Embracing change, moving with time: exploring the role of digital technologies and accelerators in promoting community oral health in Africa

**DOI:** 10.3389/froh.2025.1443313

**Published:** 2025-03-07

**Authors:** Adriano Focus Lubanga, George Kafera, Akim N. Bwanali, Yeonho Choi, Chaieun Lee, Emily Ham, Jason Y. Lee, Jaeha Chung, Jonathan Chung

**Affiliations:** ^1^ Research and Education, Clinical Research Education and Management Services, CREAMS, Lilongwe, Malawi; ^2^ Department of Clinical Services, Kamuzu Central Hospital, Lilongwe, Malawi; ^3^ School of Medicine and Oral Health, Kamuzu University of Health Sciences, Blantyre, Malawi; ^4^ Department of Clinical Services, Queen Elizabeth Central Hospital, Blantyre, Malawi; ^5^ Youth with Talents, Fairfax, VA, United States; ^6^ Research, STEM Research Institute, Fairfax, VA, United States

**Keywords:** digital technologies, oral health, Africa, oral health services, mobile health

## Background

The Sustainable Development Goals (SDGs) aim to provide a framework for addressing global environmental, political and socio-economic challenges, with the third goal focusing on ensuring healthy lives and promoting wellbeing for all (1). The third SDG emphasizes the need for achieving Universal Health Coverage (UHC) while addressing health inequities, particularly among the most vulnerable populations (1). It advocates for comprehensive access to various health services for all, including preventive, curative and rehabilitative services (1). However, five years away from the realization of the 2030 agenda, significant health inequities still exist, especially in developing countries, where the healthcare burden is characterised by infectious diseases, complicated by climate change and antimicrobial resistance (2–7). Furthermore, access to timely, adequate and quality healthcare in Africa remains a huge challenge (8).

The health inequities have not spared oral healthcare. Nearly half (44%) of the populations are affected by oral diseases in sub-Saharan Africa (SSA), yet investments in oral healthcare remain extremely low across the region (9). As of 2019, it was estimated that more than 70% of SSA countries, spend only less than a dollar per individual on treatment of oral diseases (9, 10). This greatly undermines the quality, efficiency and effectiveness of oral healthcare services provided across the region. Under-investment in healthcare greatly affects the marginalized, hard-to-reach rural communities where access to health services is usually already a big challenge (8).

Achieving global and regional goals requires addressing the existing health disparities, while ensuring that people have access to all the health services they need. This calls for investment into innovative, and less costly tools that could aid in disease promotion, training, and treatment and potentially lead to massive health gains. Digital technologies offer greater prospects and have been greatly utilized in the modern world to improve livelihood outcomes including in healthcare (11–15). Clear evidence exists that developmental accelerators such as digital technologies can bring about improved outcomes in health care and have greatly been utilized in multiple areas such as adolescent and sexual health, mental health, enhancing HIV prevention and treatment (11–15).

However, use of such technologies within the oral healthcare space has not been adequately explored. Therefore, the aim of this opinion article is to highlight how digital technologies and accelerators can be utilized to promote community oral health in Africa, thereby aiding in achieving sustainable development goals as well as Africa Agenda 2063.

## The burden of oral health diseases and the state of oral healthcare in Africa

The burden of oral diseases still remains high in Africa causing a major health concern (16). Dental carries, periodontal diseases, orofacial trauma, oral cancers, birth defects, HIV (Immunosuppression) associated oral diseases and Noma remain greatly common and affect more than 480 million (43.7%) people in Africa (16). The spatial distributions of these oral diseases skews towards the vulnerable, marginalized and poverty-stricken individuals reflecting disparities in access to oral health care services across the continent (17). The trends in the burden of oral diseases remain constant across life time, from childhood to older age, across countries with the poorer communities being greatly affected (17).

In Africa, the growing burden of oral diseases has been attributed to the ever-growing population. SSA has an annual growth rate of 2.8% and the current population is expected to double between 2022 and 2050 (17, 18). Despite the growth in population, the state of healthcare largely remains the same with most countries battling the same challenges for several decades (8, 19–21). Furthermore, the high prevalence of HIV in SSA predisposes people to oral diseases associated with immunosuppression such as oral cancers (10).

Despite oral diseases affecting a majority of population, they are highly preventable or treatable if identified in early stages. In addition, the intersection in risk factors between oral diseases and other non-communicable diseases (NCDs) offers greater prospect for maximizing on integrated approaches of health promotion at community level. Oral diseases carry similar predisposing factors to NCDs such as smoking, poor diet and alcohol consumption (22). As countries are advocating for integration of NCD counselling, education and screening at primary health care, oral health needs to be taken into consideration (9, 23). Integrating these services would not only be cost effective, but will also improve access to oral health services and eventually health outcomes. However, a clear disjuncture between oral diseases and other NCDs greatly characterizes most health care system (23, 24).

In most African countries, oral health care services are less prioritized leading to underfunding (23, 24). This affects the quality, access, and availability of oral healthcare services provided particularly among the marginalized populations. In addition to limited funding, most governments lack appropriate oral health policy documents to support the development of oral health services (10). The number of registered oral healthcare workers in Africa remains disproportional to the population. A recent estimate utilizing data from 2014 to 2019, indicates that there are only 3.3 dentist per 100,000 people. Another analysis which also assessed oral health workforce (dentists, dental assistants and therapists, and dental prosthetic technicians) across Africa also revealed notable gaps (25). Despite the growth rate of 63.6% since 2010, the study revealed that the current density of dentist (per 10,000 population) in Africa remains very low at 0.44, with marked intra-regional inequity (Seychelles, 4.297; South Sudan 0.003) (25).

Having noted the disparities in oral healthcare in Africa, and in a quest to strengthen oral health promotion, the World Health Organization (WHO) African member states convened in 2021 and resolved to set an audacious vision of achieving universal oral healthcare by 2030 (10). Since then, a comprehensive global strategy on oral health was set and adopted by African member states at the 2022 World Health Assembly (WHA).

This strategic framework provides opportune moments to spur regional commitment towards oral health. However, achieving the equitable and better access to comprehensive oral health services requires identifying more innovative ways that could help to unravel the health disparities that currently exist in Africa. Digital health technologies and accelerators remains critical towards achieving UHC.

## Digital technologies and accelerators in the healthcare space

In modern day world, digital technologies have transformed many aspects of people's lives including healthcare. Digital technologies have largely been integrated for both infectious and non-infectious diseases and have been utilized in health promotion, diagnosis and treatment (11). Digital technologies have not only transformed the way healthcare services are provided, but have also greatly improved healthcare outcomes globally. These technologies have been utilized for more than a decade to address specific challenges facing healthcare systems in Africa (26–31). No dimension of healthcare systems has been left untouched in their ability to improve care outcomes.

One outstanding transformative innovative solution is Mobile Health (mHealth). mHealth utilizes mobile phones, tablets, monitoring devices, and other wireless devices to deliver healthcare services to people, and have been greatly utilized in healthcare promotion. With the ever-growing access to mobile phones across SSA, with the number of subscribers projected to be 600 million by 2025 (32), mhealth solutions emerge as viable innovation to enhance accessibility and utilization of healthcare services among vulnerable and underserved population (11, 33).

In addition to mHealth, electronic health records (HER), health information systems (HIS), telemedicine, precision and personalized medicine, digital procurement and supply chain systems including drone delivery of medical supplies have commonly been used in public health. These technologies have been used for targeting health outcomes for which significant health disparities exist such as maternal and child health, adolescent and sexual health, common infectious diseases, and emerging NCDs (11–15).

Furthermore, lately there has been an expansion in digital space reading to development of cutting-edge robotics, artificial intelligence and deep machine learning (34–36). These advanced technologies are being explored every day and routinely being integrated into improving healthcare outcomes among people. Everyday technologies keep evolving and their application to healthcare is constantly changing.

## Digital health solutions and oral health care: bridging health disparities in oral healthcare

Oral healthcare in Africa is marked by a myriad of challenges such as limited funding, limited availability of oral healthcare worker, and limited access to oral health services particularly among vulnerable populations (10, 23, 24). Integration of digital health tools into oral healthcare can help to bridge the existing health disparities. Firstly, digital health technologies such as telemedicine and mhealth can help to solve challenges of limitations in health in oral healthcare workforce through breaking the traditional consultations. This would particularly be beneficial to neglected and socioeconomically disadvantaged groups who face the greatest need for oral health interventions, yet access to those services remains a great challenge especially in hard-to-reach areas (9, 22, 23). Mhealth has greatly been used across Africa for different diseases such as diabetes and also in monitoring maternal and child health outcomes. For instance, in Senegal, the “mDiabetes” initiative, utilized the short message service (SMS)- based technologies to deliver information related to diabetes in remote, hard-to-reach areas (31). The SMS-based approach to health promotion with regard to diabetes self-management was highly successful, demonstrating the usefulness of employing simple technologies to solve health related challenges.

Similar outcomes were also demonstrated in Ghana and Malawi where Mobile Midwife and *Chipatala cha pa phone* (health centre by phone), demonstrated greater success in delivering SMS-based health promotion messages to pregnant mothers and greatly contributed to reducing maternal and infant mortality rates in remove areas (26, 27, 31, 37).

Learning from the three case studies, it has been shown that mHealth and telemedicine is adaptable and can be applied to solve multiple healthcare challenges. In the space of oral healthcare, mHealth can be utilized to promote oral health through text-based messages about dental diseases and how to prevent them. In addition, telemedicine can be utilized to bridge the health access gap through remote consultation. Vulnerable communities would get free messages on how to maintain their oral and overall health status.

Another frontier where digital technologies can be utilized in oral health is the use of artificial intelligence (AI) for timely detection and outcome prediction in oral cancers and other diseases. Timely detection of oral malignancies remains a huge challenge, despite recent advance in technologies. Majority of people suffering from oral cancers are identified at a late stage leading to increased mortality and morbidity particularly among the vulnerable, disadvantaged populations (38, 39).

In modern day medicine, evidence continues to grow on the use of AI algorithms in promoting early and accurate diagnosis of diseases (34, 35, 40). AI algorithms have been utilized in risk stratification of stroke based on symptoms and genetic history to timely detect victims who are at greater risk of stroke (35). The algorithm demonstrated an accuracy rate as high as 87.6% in both diagnosis and prognosis prediction of at-risk patients (35). This aided in timely planning of treatment and prevention of those that demonstrated greater stroke risk.

Similarly, studies done in the United Kingdom have demonstrated greater effectiveness of AI in detection of lung cancers, with accuracy similar to those of professional radiologist (41). The AI based systems was able to accurately detect early-stage lung cancer 94 percent of the time. Timely detection led to improvement in care outcomes among people with lung cancers (42).

Further expansion into cancer detection through AI, studies have also shown that AI can diagnose colon cancer more accurately than a trained pathologist (43). Researchers collected images from more than 8,000 participants from various independent centres and used them to train a machine learning program. After thorough development of performance measurement tools, they were able to compare machine learning to real work of pathologist. Averagely average pathologist scored 0.969 for accuracy of colon cancer identification compared to 0.98 for machine learning.

With AI holding enormous promise in the field of early diagnosis and outcome prediction in diagnosing of cancers and other diseases, its applicability and adaptability to the field of oral healthcare cannot be undermined. Leveraging through sophisticated machine learning algorithms, AI can help to examine through large data sets, including patients, diagnostic images, and actual images to help in timely and accurate detection of oral cancers (35, 36). Moreover, AI can also assist oral healthcare providers with prognostic capabilities, thereby helping healthcare workers to tailor care based on individual condition and patient profile. This would help to design and provide more personalized interventions.

In a continent where access to radiologist, pathologist, dental surgeons and many other healthcare workers remains a huge problem, AI-augmented diagnostic technologies would offer a new paradigm shift in providing improved, personalized and timely health care services to the people, particularly in the field of oral health.

Africa would leverage on the existing evidence on the use of AI in improving screening and detection of oral cancers. Evidence from elsewhere clearly shows that algorithms are being developed and are proven effective (38, 40, 44). For instance, a systematic review conducted by Sanjeev et.al from Saudi Arabia, gathering nineteen articles from 2000 to 2023 on oral cancer and AI, demonstrated sensitivity and specificity of greater than 90% of AI tools in detecting oral cancers (39). The AI models' abilities to detect, categorize, and predict the occurrence of oral cancers outperformed existing clinical approaches. This demonstrates that AI is as effective as human beings in timely detection of oral cancers, thereby aiding in everting mortality and morbidity associated with late detection of oral cancers.

The digital technology space could also be utilized in training of primary healthcare workers in delivery of oral health services. Digital trainings were largely utilized during the COVID-19 pandemic when health systems faced huge disruptions in service provision (45, 46). Oral healthcare services were among the affected, with around 90% of countries responding to a WHO survey reporting a complete or partial disruption of oral health services between February and July 2020 (WHO, 2021). Having noted the disparities oral healthcare services access and limited availability of oral health service providers, WHO and the Harvard School of Dental Medicine facilitated an online training of community healthcare workers in oral healthcare. The training was piloted in four countries including Angola, Kenya, Liberia and Senegal and aimed at reinforcing the competency of primary health care workers in oral health through competency-based trainings.

The use of digital technologies including online training tools become more prominent during the COVID-19 disruptions (45, 46). Reflecting on the success of the online training implementation during the pandemic, a leaf could be borrowed to design and implement successful oral health training courses aimed at improving the state of oral health services at community level. Through this, health service providers could be trained in disease screening, risk identification, treatment and appropriate referral system.

Lastly, access to comprehensive information with regards to the burden of oral health diseases in Africa remains a huge challenge. This is further complicated by limited diagnostic capabilities as well as access to timely and affordable oral healthcare services (10, 25). Digital health solutions have widely been utilized in hospital information management systems to analyse and identify patients’ needs and preferences, thereby enhancing patient care (47–53).

Furthermore, recent development in HIS including eHealth, remote data change, mobile communication and many others have changed the narrative in the way healthcare information is handled. Improvement in information flow has led to timely follow-up and improvement in continuity of care through availability of previous patient information which can be easily accessed from anywhere and at any time (52). In the space of oral health, eHealth records and information management systems can be used in day-to-day keeping of patients records and data exchange. This would make it easy in estimating the true burden of oral diseases, their epidemiology and inform appropriate policies.

## Conclusions and recommendations for the future

Even though digital health technologies are reshaping the global healthcare space, their adaptability and applicability to solving health challenges need to be always contextualized. Countries need to develop specific policies targeting existing and emerging digital solutions that could help solve existing health disparities. Serious investment is also needed to square the digital gap between urban and rural areas. This will allow timely and wider coverage of digital services for improved oral health. Within the oral health space, there is so much opportunity to integrate digital technologies in order to overcome existing health challenges. Oral health program implementers and policy makers need to explore the various potential areas of integration, including information technology, eHealth, telemedicine and use of AI to aid timely and accurate diagnosis of oral health diseases. However, a clear consideration needs to be made to overcome barriers associated with acceptance of digital solutions and data security issues.
